# Higher visceral adiposity index was associated with an elevated prevalence of gallstones and an earlier age at first gallstone surgery in US adults: the results are based on a cross-sectional study

**DOI:** 10.3389/fendo.2023.1189553

**Published:** 2023-06-15

**Authors:** Gaopeng Zhang, Zhe Ding, Junping Yang, Tianqi Wang, Li Tong, Jian Cheng, Chao Zhang

**Affiliations:** ^1^ Department of Emergency Surgery, Hefei BOE Hospital, Hefei, China; ^2^ Department of General Surgery, First Affiliated Hospital of Anhui Medical University, Hefei, China; ^3^ Department of General Practice, Wuhu City Second People’s Hospital, Wuhu, China

**Keywords:** gallstone prevalence, VAI, cross-sectional study, age at first gallstone surgery, metabolic syndrome

## Abstract

**Objective:**

We sought to evaluate the association between visceral adiposity index (VAI) and the incidence of gallstones and the age at first gallstone surgery in adults in the United States.

**Methods:**

We selected individuals from the National Health and Nutrition Examination Survey (NHANES) database from 2017 to 2020 and evaluated the association between VAI and gallstone incidence and age at first gallstone surgery using logistic regression analysis, subgroup analysis, and dose–response curves.

**Results:**

A total of 7,409 participants aged >20 years were included in our study; 767 had a self-reported history of gallstones. After adjustment for all confounding factors, for each unit of VAI after Ln conversion, gallstone prevalence increased by 31% (OR = 1.31, 95% CI: 1.17, 1.48), while the first gallstone surgery was 1.97 years earlier (β = −1.97, 95% CI: −3.35, −0.42). The dose–response curves showed a positive correlation between VAI and gallstone prevalence. There was a negative correlation between increased VAI and age at first gallstone surgery.

**Conclusion:**

A higher VAI is positively associated with the prevalence of gallstones and may lead to an earlier age at first gallstone surgery. This is worthy of attention, although causality cannot be established.

## Introduction

Gallstones are one of the most common diseases of the digestive system worldwide and are a clear risk factor for gallbladder cancer (1, 2). Gallstones are a significant health care burden in America, affecting up to 15% of the population (3, 4). Epidemiologic evidence suggests that the prevalence of gallstones is 10% to 15% in adult Caucasians and as high as 70% in American Indians (5, 6). However, the prevalence in Asian populations is low (7, 8). Gallstones are mainly divided into cholesterol stones, melanin stones, and mixed stones, among which cholesterol stones and cholesterol components, mainly mixed stones, account for more than 80% of all stones (9, 10). In general, gallstones do not cause any symptoms, but 10% to 25% of patients may have specific symptoms such as biliary pain and acute cholecystitis, of which 1% to 2% may have major complications (1, 11, 12), causing endless pain and even being life-threatening to patients. Although previous studies have reported risk factors for gallstone formation, there is still a lack of reliable clinical indicators to prevent the occurrence of gallstones.

Current evidence suggests that non-modifiable risk factors for the development of gallstones include race, female gender, pregnancy, and age over 40 years, with a 4- to 10-fold increased risk of gallbladder disease (3, 4). Women of childbearing age are about twice as likely as men to develop gallstone disease (4). The most important modifiable risk factor for the development of gallstones is metabolic syndrome (13). This includes obesity, dyslipidemia, type 2 diabetes mellitus, insulin resistance, etc. Obesity, especially abdominal obesity (about 25% of the population has abdominal obesity), is closely related to the occurrence of gallstones (3). Some studies have shown that obesity is a risk factor for gallstones (14–16), and the incidence of gallstones has increased 1.63 times for every five-unit increase in body mass index (15). Although obesity is strongly associated with the occurrence of gallstones, reliable obesity indicators to predict and assess the risk of gallstones are severely lacking.

As the main form of energy storage in the human body, adipose tissue is the regulator of lipid metabolism and glucose balance (17). Studies have shown that visceral adipose tissue is more closely associated with metabolic diseases such as hypertension, diabetes, and cardiovascular disease than is subcutaneous fat (18–20). The Visceral Fat Index (VFI), an indicator of abdominal fat distribution and adipose tissue function that indirectly expresses visceral fat function based on waist circumference, BMI, triglycerides, and HDL cholesterol, is a novel specificity index (21). It has a lower practice cost and better applicability than the traditional methods used to assess body fat content and distribution (magnetic resonance imaging, computed tomography, dual-energy X-ray absorptiometry, etc.). Some scientists believe that VAI has higher sensitivity and specificity than traditional body parameters such as body mass index and waist circumference. Studies have shown that the clinical application of VAI can significantly improve the risk assessment of obesity-related cardiovascular diseases (21–23). In conclusion, we speculate that there is a relationship between VAI and the occurrence of gallstones, and therefore, in this study, we aimed to evaluate the value of VAI in the occurrence of gallstones in the US adult population.

## Materials and methods

### Study design and participants

**Figure 1:**
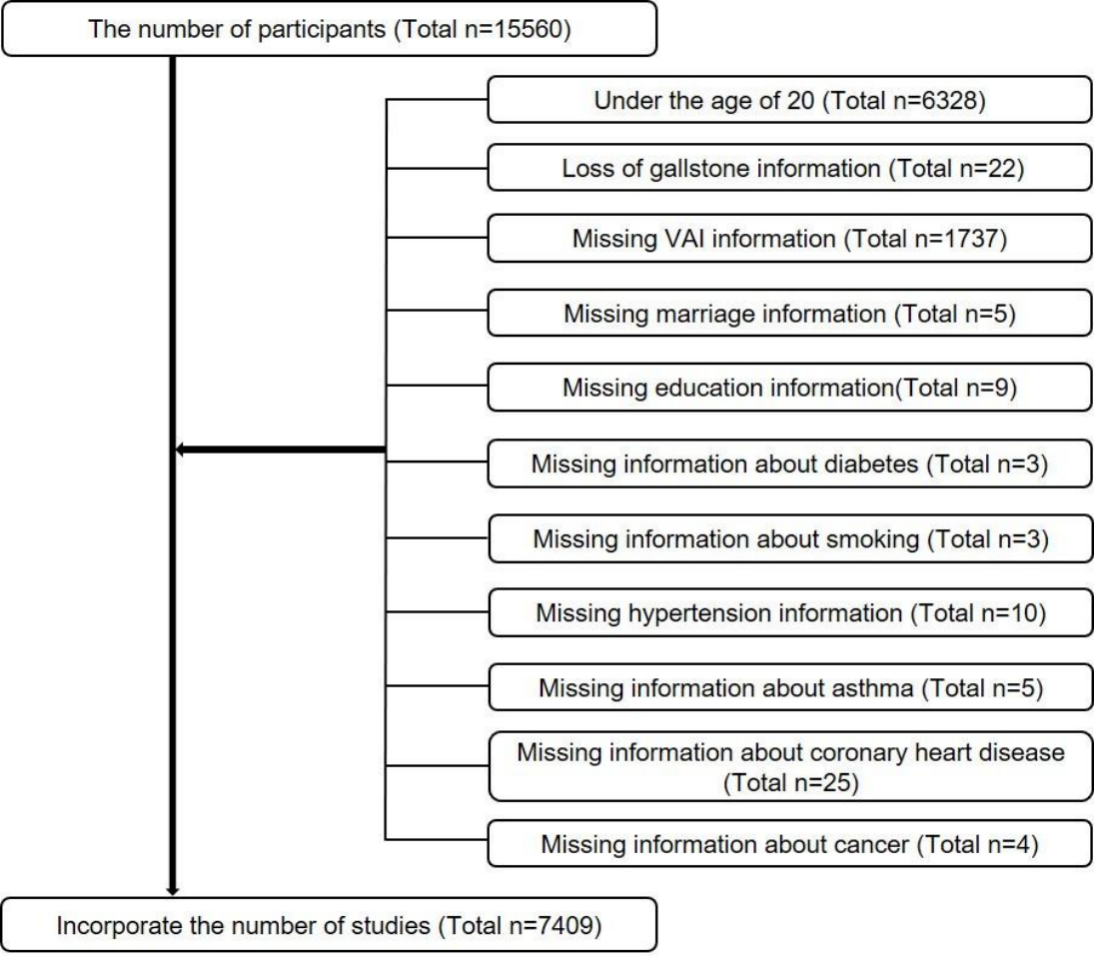
The participants selection flow chart.

The baseline clinical data evaluated in this study were from the 2017–2020 NHANES. We retained information on participants who explicitly answered whether they had gallstones and their age at first gallstone surgery. A total of 15,560 individuals completed the questionnaire. Exclusion criteria were as follows (**Figure 1**). Finally, a total of 7,409 cases were included in this study, including 767 self-reported histories of gallstones.

### Collection and definition of data

The VAI was designed as an exposure variable and was calculated using the following sex-specific equations, where the units for WC, BMI, TG, and HDL are cm kg/m^2^ and mmol/L: males: VAI = [WC/[39.68 + (1.88 × BMI)]) × (TG/1.03) × (1.31/HDL); women: VAI = [WC/[36.58 + (1.89 × BMI)]) × (TG/0.81) × (1.52/HDL)]. The concentrations of triglycerides and fasting blood glucose were determined enzymatically using an automated biochemical analyzer. Serum triglyceride concentrations were measured using a Roche Modular P and a Roche Cobas 6000 chemistry analyzer. A questionnaire was used to assess the presence of gallstones and age at first gallstone surgery. The development of gallstones and age at first gallstone surgery were used as outcome variables.

Potential covariates that could confound the association between VAI and gallstones were summarized in the multivariable-adjusted model. Covariates in our study included sex (male/female), age (years), ethnicity, education level, poverty income ratio (PIR), marital status (married or living with a partner/single), alcohol consumption (drinking alcohol/not drinking alcohol), physical activity (vigorous/moderate/less than moderate), cholesterol level (mg/dl), smoking status, hypertension, diabetes, asthma, hypertension, cancer, and dietary intake factors, including energy intake, fat intake, sugar intake, and water intake. In 2017–2020, all participants had 24-hour dietary recalls; our analysis will use the average consumption rate for the two recalls. All detailed measurement procedures using the study variables are publicly available at www.cdc.gov/nchs/nhanes/. All NHANES protocols were conducted in accordance with the US Department of Health and Human Services (HHS) Human Research Subjects Protection Policy and were reviewed and standardized annually by the NCHS Research Ethics Review Board. All subjects participating in the study signed an informed consent form. All data in this study were freely released by NHANES without additional permission or ethical review.

### Statistical methods

The NHANES sampling weights, stratification, and clustering provided in the study were applied to all statistical analyses to illustrate the complex, multistage sampling design used to select a representative non-institutionalized U.S. population. Continuous variables were expressed as weighted survey means and 95% CIs, and categorical variables were expressed as weighted survey means and 95% CIs. As VAI have skewed distributions, LN transformations convert them into normal distributions. We first screened all covariates for VIF collinearity and removed them if VIF values greater than 5 were considered collinear. As VAI have skewed distributions, LN transformations convert them into normal distributions. According to the guidelines (24, 25), the multiple logistic regression model was used to examine the relationship between VAI, different VAI tiles, gallstone prevalence, and age of first gallstone surgery in the three different models. Model 1 was unadjusted for covariates. Model 2 was adjusted for sex, age, ethnicity, marital status, and education level. Model 3 was adjusted for all variables. Smoothing curve fitting (penalty spline method) and generalized additive model (GAM) regression were performed to further evaluate the relationship between VAI and gallstone prevalence and age at first gallstone surgery. The inflection point values were obtained by the natural ratio test when the presence of nonlinear relationships was determined. Multiple regression analysis was then performed, stratified by sex, age, race, hypertension, and diabetes. A p <0.05 was considered statistically significant. All analyses were performed using Empower software (www.empowerstats.com; X&Y Solutions, Inc., Boston, MA, USA) and R version 4.0.2 (http://www.R-project.org, The R Foundation).

## Results

### Participant characteristics

The baseline demographic characteristics of the included participants are shown in **Table 1**. Ln (VAI) was 2.69 (2.37, 3.01) in the gallstone group, higher than the normal group of 2.17 (2.06, 2.27), P <0.01.

**Table 1:** Baseline characteristics of participants, weighted.

Characteristic	Non-stone formers (n = 6,642)	Stone formers (n = 767)	*P-*value
Age (years)	47.17 (46.01, 48.33)	56.67 (55.38, 57.97)	<0.0001
Serum Creatinine (mg/dl)	0.88 (0.87, 0.89)	0.86 (0.83, 0.88)	0.0688
Ln (VAI)	2.17 (2.06, 2.27)	2.69 (2.37, 3.01)	0.0062
Race (%)			0.0004
Mexican American	8.53 (6.34, 11.37)	7.86 (5.54, 11.02)	
White	70.50 (65.96, 74.66)	77.36 (71.74, 82.13)	
Black	11.13 (8.45, 14.51)	6.59 (4.83, 8.95)	
Other Race	9.85 (7.94, 12.15)	8.19 (5.49, 12.06)	
Physical Activity (%)			<0.0001
Vigorous	50.01 (47.99, 52.04)	33.53 (28.80, 38.62)	
Moderate	27.89 (25.63, 30.27)	36.49 (31.01, 42.34)	
Never	22.09 (20.66, 23.59)	29.98 (25.58, 34.77)	
Marital Status (%)			0.0001
Cohabitation	62.39 (59.70, 65.01)	64.97 (59.22, 70.31)	
Solitude	17.63 (16.24, 19.12)	22.89 (18.75, 27.63)	
Never married	19.98 (17.93, 22.20)	12.14 (9.01, 16.18)	
Alcohol (%)			<0.0001
Yes	13.93 (12.62, 15.36)	26.76 (21.67, 32.55)	
No	76.61 (74.85, 78.28)	62.62 (58.15, 66.87)	
Unclear	9.46 (8.24, 10.84)	10.63 (7.89, 14.17)	
High Blood Pressure (%)			<0.0001
Yes	30.31 (28.09, 32.64)	48.46 (42.31, 54.66)	
No	69.69 (67.36, 71.91)	51.54 (45.34, 57.69)	
Diabetes (%)			<0.0001
Yes	10.05 (9.14, 11.04)	20.20 (17.43, 23.29)	
No	89.95 (88.96, 90.86)	79.80 (76.71, 82.57)	
Asthma (%)			0.0749
Yes	14.84 (13.52, 16.26)	18.29 (14.43, 22.90)	
No	85.16 (83.74, 86.48)	81.71 (77.10, 85.57)	
Coronary Heart Disease (%)			0.0001
Yes	3.72 (2.53, 5.42)	7.31 (5.21, 10.17)	
No	96.28 (94.58, 97.47)	92.69 (89.83, 94.79)	
Cancers (%)			<0.0001
Yes	10.17 (9.26, 11.17)	17.95 (13.91, 22.84)	
No	89.83 (88.83, 90.74)	82.05 (77.16, 86.09)	
Smoked (%)			0.0402
Yes	42.24 (40.35, 44.15)	47.76 (41.31, 54.29)	
No	57.76 (55.85, 59.65)	52.24 (45.71, 58.69)	
PIR			0.0021
<1.3	16.26 (14.79, 17.85)	15.67 (11.87, 20.41)	
≥1.3–<3.5	30.06 (27.38, 32.88)	39.67 (32.55, 47.25)	
≥3.5	42.81 (39.69, 45.99)	35.55 (31.01, 40.36)	
Unclear	10.86 (9.32, 12.63)	9.11 (6.65, 12.35)	
Total Sugar (%)			0.0185
Lower	41.96 (39.96, 43.99)	39.96 (35.22, 44.91)	
Higher	40.18 (38.56, 41.83)	46.16 (41.43, 50.96)	
Unclear	17.86 (16.24, 19.60)	13.88 (11.48, 16.68)	
Total Kcal (%)			0.0003
Lower	38.50 (36.61, 40.43)	47.62 (42.52, 52.77)	
Higher	43.64 (42.30, 44.99)	38.50 (33.10, 44.21)	
Unclear	17.86 (16.24, 19.60)	13.88 (11.48, 16.68)	
Total Fat (%)			0.0004
Lower	38.05 (36.65, 39.46)	47.02 (41.76, 52.35)	
Higher	44.09 (42.64, 45.55)	39.10 (33.68, 44.80)	
Unclear	17.86 (16.24, 19.60)	13.88 (11.48, 16.68)	
Total Water (%)			0.0208
Lower	35.20 (33.30, 37.15)	41.25 (36.63, 46.04)	
Higher	46.94 (44.73, 49.16)	44.87 (39.30, 50.57)	
Unclear	17.86 (16.24, 19.60)	13.88 (11.48, 16.68)	

Data of continuous variables are shown as survey-weighted mean (95% CI), P-value was calculated by survey-weighted linear regression. Data of categorical variables are shown as survey-weighted percentage (95% CI), P-value was calculated by survey-weighted Chi-square test.

### Logistic regression results between VAI and gallbladder stones

VIF collinearity screened all covariates with VIF values <5, and all variables were included in the final regression model. For gallstones, a positive correlation was observed between VAI and gallstones. This positive correlation remained stable in the fully adjusted model (model 3) (OR = 1.31, 95% CI: 1.17, 1.48), indicating that each unit increase in Ln-converted VAI was associated with a 31% increase in gallstone prevalence. We also converted VAI from continuous to categorical variables (tertiles) for sensitivity analysis. A significant 0.8-fold increase in gallstone incidence was observed in tertile 3 compared with the lowest VAI tertile (tertile 1) (OR = 1.80, 1.45, 2.25) (**Table 2**).

**Table 2:** Logistic regression analysis between VAI with gallbladder stone prevalence.

Characteristic	Model 1 OR (95% CI)	Model 2 OR (95% CI)	Model 3 OR (95% CI)
Ln (VAI)	1.52 (1.37, 1.68)	1.41 (1.26, 1.57)	1.31 (1.17, 1.48)
Categories			
Tertile 1	1	1	1
Tertile 2	2.02 (1.64, 2.49)	1.73 (1.40, 2.14)	1.61 (1.30, 2.00)
Tertile 3	2.52 (2.06, 3.09)	2.08 (1.68, 2.56)	1.80 (1.45, 2.25)

Model 1 = no covariates were adjusted.

Model 2 = Model 1 + age, gender, race education, and marital status were adjusted.

Model 3 = Model 2 + diabetes, blood pressure, education, PIR, asthma, total water, total kcal, total fat, total sugar, smoked, physical activity, alcohol use, serum creatinine, serum cholesterol, cancers, and CVD were adjusted.

### VAI’s dose–response and threshold effect on gallbladder stone prevalence

**Figure 2:**
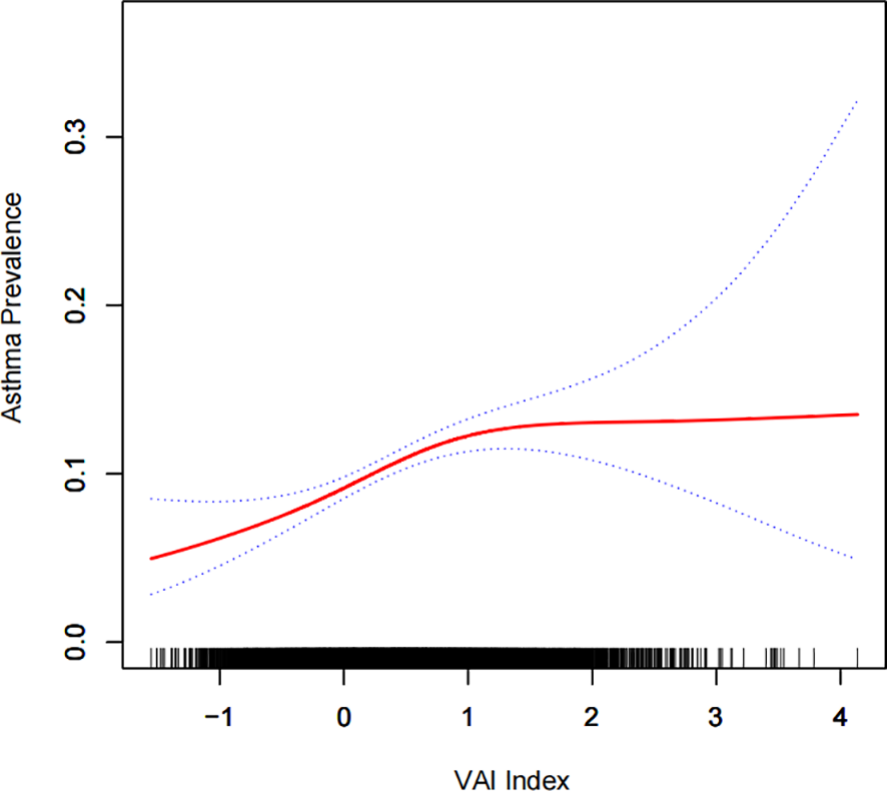
Density dose-response relationship between the Ln (VAI) index and gallstone prevalence. The area between the upper and lower dashed lines is represented as the 95% CI. Each point shows the magnitude of the Ln (VAI) index and is connected to form a continuous line. Adjusted for all covariates except the effect modifier.

A generalized additive model and smooth curve fitting were used to further explore the relationship between Ln (VAI) and gallstone incidence. Our results indicated a non-linear relationship between Ln (VAI) and gallstone incidence (**Figure 2**; **Table 3**). Considering the effect of the saturation threshold between them, the likelihood natural ratio test found the best Ln-transformed VAI threshold at 0.6.

**Table 3:** Two-piecewise linear regression and logarithmic likelihood ratio test explained the threshold effect analysis of VAI with gallbladder stone prevalence.

Ln (VAI)	ULR Test	PLR Test	LRT test
	OR (95% CI)	OR (95% CI)	P-value
<0.6 umol/L	1.28 (1.14, 1.44)	1.72 (1.34, 2.21)	0.006
≥0.6 umol/L		1.02 (0.84, 1.25)	

ULR, univariate linear regression; PLR, piecewise linear regression; LRT, logarithmic likelihood ratio test, statistically significant: p <0.05.

### Subgroup analysis

Subgroup analyses were performed to assess the robustness of the association between Ln (VAI) and gallstone incidence. The results were as follows (**Table 4**):

● Female group (OR = 1.39, 95% CI: 1.20, 1.62),
● Age group 20–39 years (OR = 1.62, 95% CI: 1.21, 2.16),
● Age group 40–59 years (OR = 1. 26, 95% CI: 1.04, 1.54),
● White group (OR = 1.32, 95% CI: 1.12, 1.56),
● Other population groups (OR = 1.61, 95% CI: 1.19, 2.18),
● Hypertension population group (OR = 1. 27, 95% CI: 1.07, 1.50),
● Non-hypertensive population group (OR = 1.33, 95% CI: 1.12, 1.57),
● Diabetic population group (OR = 1.31, 95% CI: 1.02, 1.67),
● Non-diabetic population group (OR = 1.31, 95% CI: 1.15, 1.50).

**Table 4:** Subgroup analysis between VAI with gallbladder stone prevalence.

Characteristic	Model 1 OR (95% CI)	Model 2 OR (95% CI)	Model 3 OR (95% CI)
Stratified by age (years)	Non-adjusted	Adjust I	Adjust II
20–39	1.71 (1.37, 2.14)	1.95 (1.50, 2.52)	1.62 (1.21, 2.16)
40–59	1.37 (1.15, 1.62)	1.37 (1.14, 1.65)	1.26 (1.04, 1.54)
60–80	1.44 (1.24, 1.68)	1.30 (1.10, 1.52)	1.19 (0.99, 1.42)
Stratified by gender	Non-adjusted	Adjust I	Adjust II
Male	1.27 (1.07, 1.50)	1.27 (1.06, 1.54)	1.22 (0.99, 1.50)
Female	1.70 (1.49, 1.93)	1.53 (1.33, 1.75)	1.39 (1.20, 1.62)
Stratified by race	Non-adjusted	Adjust I	Adjust II
Mexican American	1.16 (0.89, 1.52)	1.04 (0.75, 1.43)	1.07 (0.75, 1.51)
White	1.49 (1.30, 1.72)	1.45 (1.25, 1.68)	1.32 (1.12, 1.56)
Black	1.67 (1.32, 2.12)	1.48 (1.15, 1.91)	1.31 (0.99, 1.72)
Other Race	1.53 (1.17, 2.00)	1.52 (1.15, 2.01)	1.61 (1.19, 2.18)
Stratified by hypertension	Non-adjusted	Adjust I	Adjust II
YES	1.40 (1.21, 1.62)	1.31 (1.12, 1.54)	1.27 (1.07, 1.50)
NO	1.48 (1.28, 1.70)	1.38 (1.18, 1.62)	1.33 (1.12, 1.57)
Stratified by diabetes	Non-adjusted	Adjust I	Adjust II
YES	1.40 (1.14, 1.72)	1.29 (1.03, 1.63)	1.31 (1.02, 1.67)
NO	1.44 (1.28, 1.61)	1.34 (1.18, 1.53)	1.31 (1.15, 1.50)

Model 1 = no covariates were adjusted.

Model 2 = Model 1 + age, gender, race education, and marital status were adjusted.

Model 3 = adjusted for all covariates except effect modifier.

### Elevated VAI may be associated with earlier age at first gallbladder stone surgery

The unit of Ln (VAI) was 1.97 years earlier (β = −1.97, 95% CI: −3.35, −0.42) in fully adjusted model 3 (**Table 5**).

**Table 5:** Analysis between VAI with age at the first gallbladder stone operation.

Characteristic	Model 1 β (95% CI)	Model 2 β (95% CI)	Model 3 β (95% CI)
Ln (VAI)	−0.65 (−2.30, 1.01)	−0.94 (−2.52, 0.64)	−1.97 (−3.53, −0.42)

Model 1 = no covariates were adjusted.

Model 2 = Model 1 + gender, race education, and marital status were adjusted.

Model 3 = Model 2 + diabetes, blood pressure, education, PIR, asthma, total water, total kcal, total fat, total sugar, smoked, physical activity, alcohol use, serum creatinine, serum cholesterol, cancers, and CVD were adjusted.

### Analysis of the dose–response and threshold effects of VAI on age at first gallbladder stone surgery

**Figure 3:**
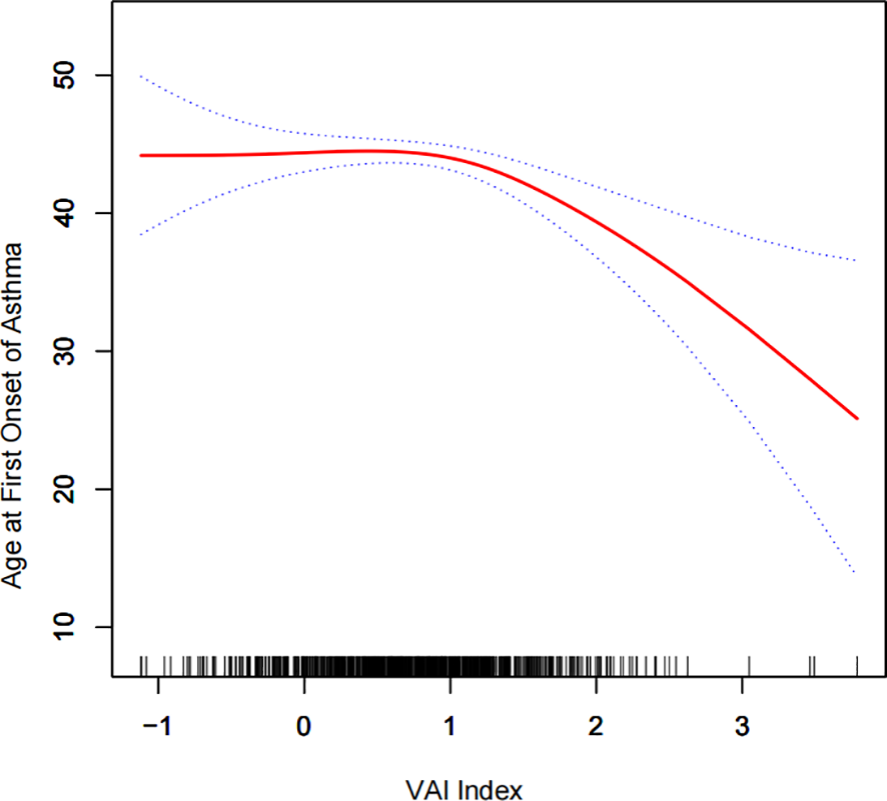
Density dose–response relationship between Ln (VAI) index and age at first gallstone surgery. The area between the upper and lower dashed lines is represented as the 95% CI. Each point shows the magnitude of the VAI and is connected to form a continuous line. Adjusted for all covariates except the effect modifier.

To further investigate the relationship between VAI and age at first gallstone surgery, a generalized additive model and smooth curve fitting were used. Our results indicated a negative nonlinear correlation between VAI and age at first gallstone surgery (**Figure 3**). The threshold for the effect of VAI on age at first gallstone surgery after Ln conversion was 0.8, according to the similar natural ratio test (**Table 6**).

**Table 6:** Two-piecewise linear regression and logarithmic likelihood ratio test explained the threshold effect analysis of VAI with age at the first gallbladder stone operation.

Ln (VAI)	ULR Test	PLR Test	LRT test
	OR (95% CI)	OR (95% CI)	P-value
<0.8 umol/L	−1.97 (−3.53, −0.42)	0.55 (−1.55, 2.66)	<0.001
≥0.8 umol/L		−8.59 (−12.64, −4.54)	

ULR, univariate linear regression; PLR, piecewise linear regression; LRT, logarithmic likelihood ratio test, statistically significant: p <0.05.

## Discussion

To our knowledge, this study is the first comprehensive analysis of the association between VAI and gallstones. Two-cycle population study (2017–2020) based on the NHANES database. The results showed a positive association between VAI and gallstone incidence, with each unit increase in VAI increasing the incidence by 31% (OR = 1.31, 95% CI: 1.17, 1.48). Furthermore, we found that increased VAI was associated with an earlier age of first gallstone surgery, with each unit increase in VAI leading to an earlier age of 1.97 years (beta = −1.97, 95% CI: −3.35, −0.42). At the same time, we converted VAI from a continuous variable to a categorical variable (tertiles) and found that the probability of gallstones in individuals with the highest VAI was 0.8 times higher than the lowest quantile, and the prevalence of individuals with the highest VAI was 0.61 times higher than the lowest quantile. Furthermore, in the stratified analysis, we found a higher risk of gallstone disease in the female group (OR = 1.39, 95% CI: 1.20, 1.62), 20–39 (OR = 1.39, 95% CI: 1.04, 1.54:1.32, 1.62), and other population groups (OR = 1.56) (OR = 1.61, 95% CI: 1.19, 2.18). Moreover, the incidence of gallstones was positively associated with increased VAI in hypertensive, non-hypertensive, diabetic, and non-diabetic groups. These results strongly support the value of VAI as a predictor of gallstone development.

The formation of gallstones results from a combination of genetic factors and environmental stimuli. In particular, dietary factors may directly or indirectly lead to the occurrence of gallstones, such as overweight, obesity (26), insulin resistance (13), and metabolic syndrome (27). While VAI is a more specific index than BMI and triglycerides, our results suggest that the female group and white group increased VAI levels in the age group, 60-year-old group, hypertensive/non-hypertensive group, and diabetic/non-diabetic group after adjusting for all confounding factors. Numerous previous studies have shown that women have a higher prevalence of gallstones than men (28, 29). Estrogen increases gallstone formation by increasing hepatic cholesterol synthesis and secretion and decreases bile salt synthesis by upregulating estrogen receptor 1 and G protein-coupled receptor 30 (30). Progestational sex hormones are thought to put women at greater risk for disease, and estrogen can increase the release of cholesterol into the bile, leading to cholesterol saturation and gallbladder stones (31). This may partly explain why the prevalence of gallstones was higher in women than in men. In addition, the results of a Korean study showed that high VAI levels were associated with a high prevalence of asymptomatic cerebral infarction in a healthy population, especially in the female population (32). Although this study was not related to gallstones, it also shows the reliability of our study results.

In the United States, the prevalence of gallstones was 16.6% in white women and 8.6% in men, compared with 13.9% in black women and 5.3% in men (7, 33). In developed countries, 10% to 15% of white adults have gallstones, compared with a lower incidence of gallstones in the black population (5). These results suggest that white Americans have a higher prevalence of gallstones, which may explain our findings. In fact, age as a risk factor for gallstones is controversial. Gallstones were once thought to be associated with pigment stones that occur only in cases of hemolysis, but they are increasingly common in children (34). The incidence of gallstones increases with age and rises significantly after the age of 40, with a 4- to 10-fold increase in the elderly (5). Although gallstones are usually clinically asymptomatic, symptoms and serious complications increase with age, leading to cholecystectomy in over 40% of people over 40 years of age (35). The high prevalence of gallstones may occur in older women (70 to 79 years): 57% have a history of cholecystectomy or current gallstones (8). Therefore, the influence of age on gallstone formation should be further investigated.

In hepatocytes, insulin resistance induces abnormal expression of the transcription factor forkhead box protein O1 (FOXO1) through the ABCG5 and ABCG8 genes to promote cholesterol secretion (36). This mechanism may explain the high prevalence of gallstones in diabetic patients, which is consistent with our findings. Interestingly, our results showed that elevated VAI levels were also positively associated with increased gallstone incidence in the non-diabetic group, with its OR value equal to the statistical results in the diabetic group. In a cross-sectional study by Ali, 204 patients with gallstones were included, of whom 74 were diabetic, 79 were non-diabetic, 51 were pre-diabetic, one had well-controlled diabetes, and one had poorly controlled diabetes (37). The results indicate that diabetes is a risk factor for gallstones, and non-diabetes also seem to be associated with the occurrence of gallstones. Furthermore, in a Korean study, metabolic syndrome was associated with gallstone development in non-hypertensive and non-diabetic patients (38). The study by Chen (39) showed that the elevated metabolic syndrome specific index was associated with increased asthma prevalence in non-hypertensive and non-diabetic populations, while Shen’s study found a correlation between the METS-IR index and the prevalence of kidney stones in non-hypertensive and non-diabetic populations (40). Our results showed a strong correlation between the prevalence of gallstones in the non-hypertensive group (OR = 1.33, 95% CI: 1.12, 1.57) and in the hypertensive group (OR = 1.27, 95% CI: 1.07, 1.50). The above two research topics are not specific to gallstones. However, they also demonstrate the reliability of our experimental results. In addition, we found an interesting result: for every 1 unit of VAI, the age of first gallstone surgery was 1.97 years earlier, and the smooth curve fitting showed a non-linear negative correlation between VAI and the age of first gallstone surgery. This result has not been reported before, and if it is confirmed by more studies, it reminds us that more attention and management of VAI at a young age will help to reduce the occurrence of gallstones.

Our study has several strengths. First, the study participants in NHANES are a representative sample of the U.S. who strictly followed the carefully designed study protocol with strict quality control and assurance to ensure that our conclusions are reliable. Second, we adjusted for confounding variables and performed subgroup analysis to ensure that our results apply to a broader range of individuals. However, our study has several limitations. First, our study was a cross-sectional study, which did not allow us to clarify the causal relationship between VAI and gallstones. Second, all the survey data were based on questionnaires, and there may be recall bias. Despite these limitations, this paper is the first to reveal the relationship between VAI and the prevalence of gallstones.

## Conclusion

This study showed an association between the modifiable risk factor VAI, the prevalence of gallbladder stones, and age at first gallbladder stone surgery. A higher VAI was associated with an increased prevalence of gallbladder stones and an earlier age for first gallbladder stone surgery. Our findings suggest that weight control and a healthy lifestyle may improve or reduce the occurrence of gallbladder stones, and although the causal relationship between the two cannot be clearly established, it is still of interest.

## Data availability statement

The datasets presented in this study can be found in online repositories. The names of the repository/repositories and accession number(s) can be found in the article/supplementary material.

## Ethics statement

The NCHS Research Ethics Review Committee approved the NHANES survey protocol. The patients/participants provided their written informed consent to participate in this study.

## Author contributions

GZ and ZD: Conceptualization, methodology, and software. JY, TW, and LT: Data curation and writing original draft. JC and CZ: Writing—review and editing. All authors contributed to the article and approved the submitted version.
